# Supplementary material for: Integration of single-cell and spatial transcriptomics by SEU-TCA reveals the spatial origin of early cardiac progenitors
Source: Genome Biol. 2025 Jun 10;26:158. doi: 10.1186/s13059-025-03633-3 (PMC12150484; doi:10.1186/s13059-025-03633-3)
Supplement: Supplementary file 1 — Additional file 1. Supplementary figures. [file 13059_2025_3633_MOESM1_ESM.docx]

**ADDITIONAL FILE 1**

**Integration of single-cell and spatial transcriptomics by SEU-TCA reveals the spatial origin of early cardiac progenitors**

Jingjing He^1, 8^, Yi Yang^1, 2, 8^, Rui Jiang^3, 8^, Yanying Zheng^1, 8^, Xianfa Yang^4^, Xu Jiang^1^, Xin Xue^1^, Zhongzhou Yang^5^, Naihe Jing^4^, Hailong Cao^1, *^, Zhuojuan Luo^1,6, *^, Ke Wei^3, *^, Peng Xie^7, *^, Chengqi Lin^1, 2, 6, *^

^1^Department of Cardiac Surgery, Zhongda Hospital, Key Laboratory of Developmental Genes and Human Disease, School of Life Science and Technology, Southeast University, Nanjing, China.
^2^Co-innovation Center of Neuroregeneration, Nantong University, Nantong, China.

^3^Institute for Regenerative Medicine, State Key Laboratory of Cardiology and Medical Innovation Center, Shanghai East Hospital, Shanghai Key Laboratory of Signaling and Disease Research, Frontier Science Center for Stem Cell Research, School of Life Sciences and Technology, Tongji University, Shanghai, China.
^4^Guangzhou Laboratory, Guangzhou, China.

^5^State Key Laboratory of Pharmaceutical Biotechnology, Department of Cardiology, Nanjing Drum Tower Hospital, The Affiliated Hospital of Nanjing University Medical School and MOE Key Laboratory of Model Animal for Disease Study, Model Animal Research Center, Nanjing University, Nanjing, China.

^6^Shenzhen Research Institute, Southeast University, Shenzhen, China. School of Biological Science & ^7^Medical Engineering, Southeast University, Nanjing, China.

^8^These authors contributed equally to this work

*To whom correspondence should be addressed at:

Chengqi Lin, PhD, Email: [cqlin@seu.edu.cn](mailto:cqlin@seu.edu.cn)

Peng Xie, PhD, Email: pengx@seu.edu.cn

Ke Wei, PhD, Email:  kewei@tongji.edu.cn

Zhuojuan Luo, PhD, Email: [zjluo@seu.edu.cn](mailto:zjluo@seu.edu.cn)

Hailong Cao, PhD, Email: shuqu_1982@sina.com

**Supplementary Figures**

**Fig S1.** The performance evaluation results of different integration methods on the human heart dataset.

**Fig S2.** Kernel function and parameter selection.

**Fig S3.** Computation time and memory usage for each method on the human heart dataset.

**Fig S4.** The performance evaluation results of different integration methods on the mouse olfactory bulb dataset.

**Fig S5.** The performance evaluation results of different integration methods on the human pancreatic ductal adenocarcinoma dataset.

**Fig S6.** Validation for SEU-TCA model using GEO-seq and scRNA-seq data.

**Fig S7.** The performance evaluation results of different integration methods on E7.5 GEO-seq and scRNA-seq data of the mouse embryo.

**Fig S8.** Mesodermal data exhibited a distribution along the P-D and A-P developmental axes after SEU-TCA alignment.

**Fig S9.** Inference of the spatial locations of cell types for mesoderm.

**Fig S10.** Consistency of cell type-specific regulon activity with its gene expression pattern.

**Fig S11.** Regulon specificity score for each spatial location.

**Fig S12.** WOT tracing analysis of JCF/pSHF/aSHF lineage.

**Fig S13.** Markers expression during the mesodermal lineage.

**Fig S14.** Specific spatial regulon in JCF/aSHF/pSHF lineages.

**Fig S15**. Expression patterns of *Irx1*/*3*/*5* in the aSHF lineage.

**Fig S16.** Generation of the *Irx1*-lineage and *Irx1* CKO mice.

**Fig S1. The performance evaluation results of different integration methods on the human heart dataset.**


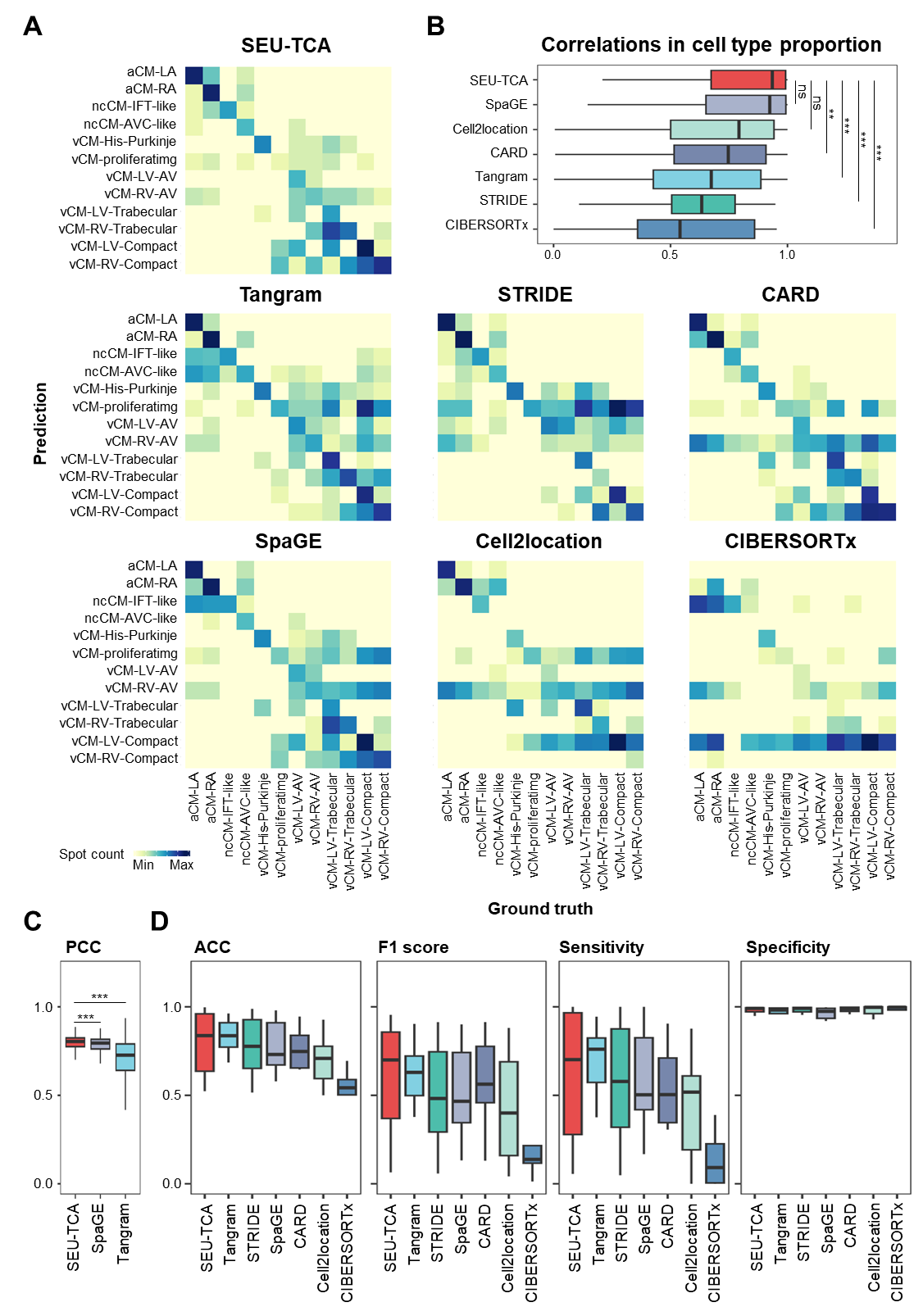


**(A)** The confusion matrix reflects the consistency between the prediction and the truth for the cell type assignment of pseudo-bulk spot data. The color is normalized by spot count. **(B)** Correlations in cell type proportion across each spot between predictions inferred by different methods and truth prior to gridding. Statistical comparisons were conducted using pairwise t-tests, and p-values were adjusted for multiple testing using the FDR method. Significance levels are denoted by asterisks: ***: p < 0.001; **: p < 0.01; ns: p > 0.05 **(C)** PCC performance of different methods on the human heart dataset. Each value represents the correlation between spot-cell pairs predicted by these three methods at the expression level of top 50 genes for each cell type. 95% confidence intervals of mean PCC values for each method were as follows: SEU-TCA ([0.785,0.788]), SpaGE ([0.771,0.774]), and Tangram ([0.703,0.707]). The same statistical methods as described in (B) were used for the analysis. **(D)** ACC, F1 score, Sensitivity, and Specificity performances of different methods on the human heart dataset. Each value represents the comparison between the predicted dominant cell type across different anatomical layers and the ground truth. The methods are ordered based on the median accuracy values, sorted from highest to lowest.

**Fig S2. Kernel function and parameter selection.**

**
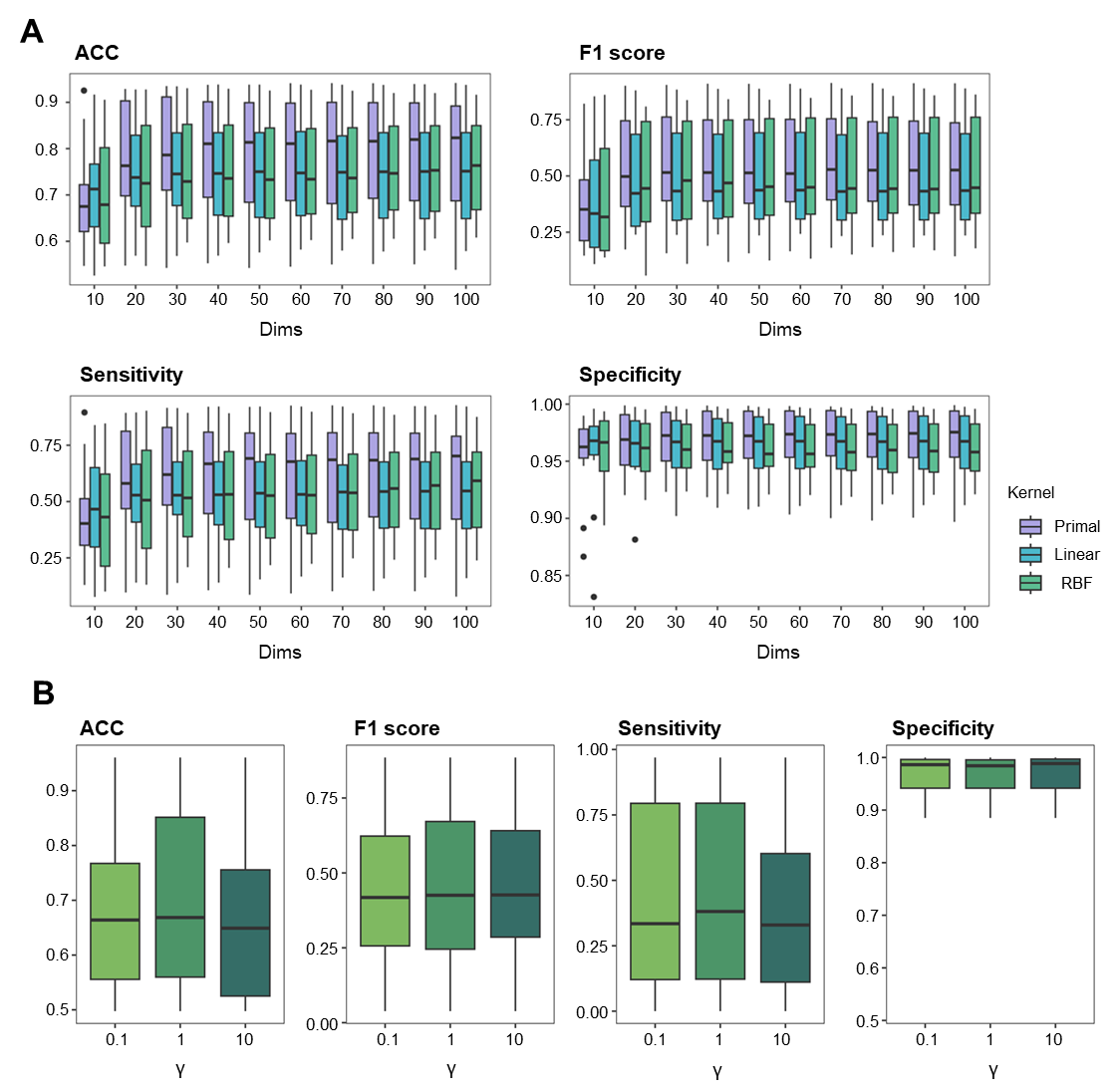
**

**(A)** Performance tests on the human heart dataset using alignment dimensions ranging from 10 to 100 under the three kernel types (Primal, Linear, RBF). **(B)** Performance tests on the human heart dataset using different bandwidth parameter (γ) for the RBF kernel.

**Fig S3. Computation time and memory usage for each method on the human heart dataset.**

**
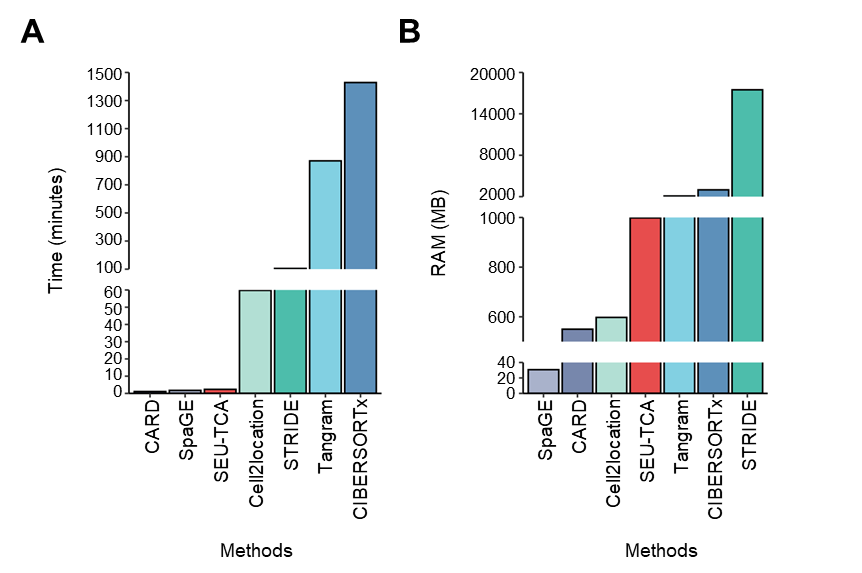
**

**(A)** Computation time (minutes) for each method on the human heart dataset. **(B)** Computation memory usage (MB) for each method on the human heart dataset.

**Fig S4. The performance evaluation results of different integration methods on the mouse olfactory bulb dataset.**

**
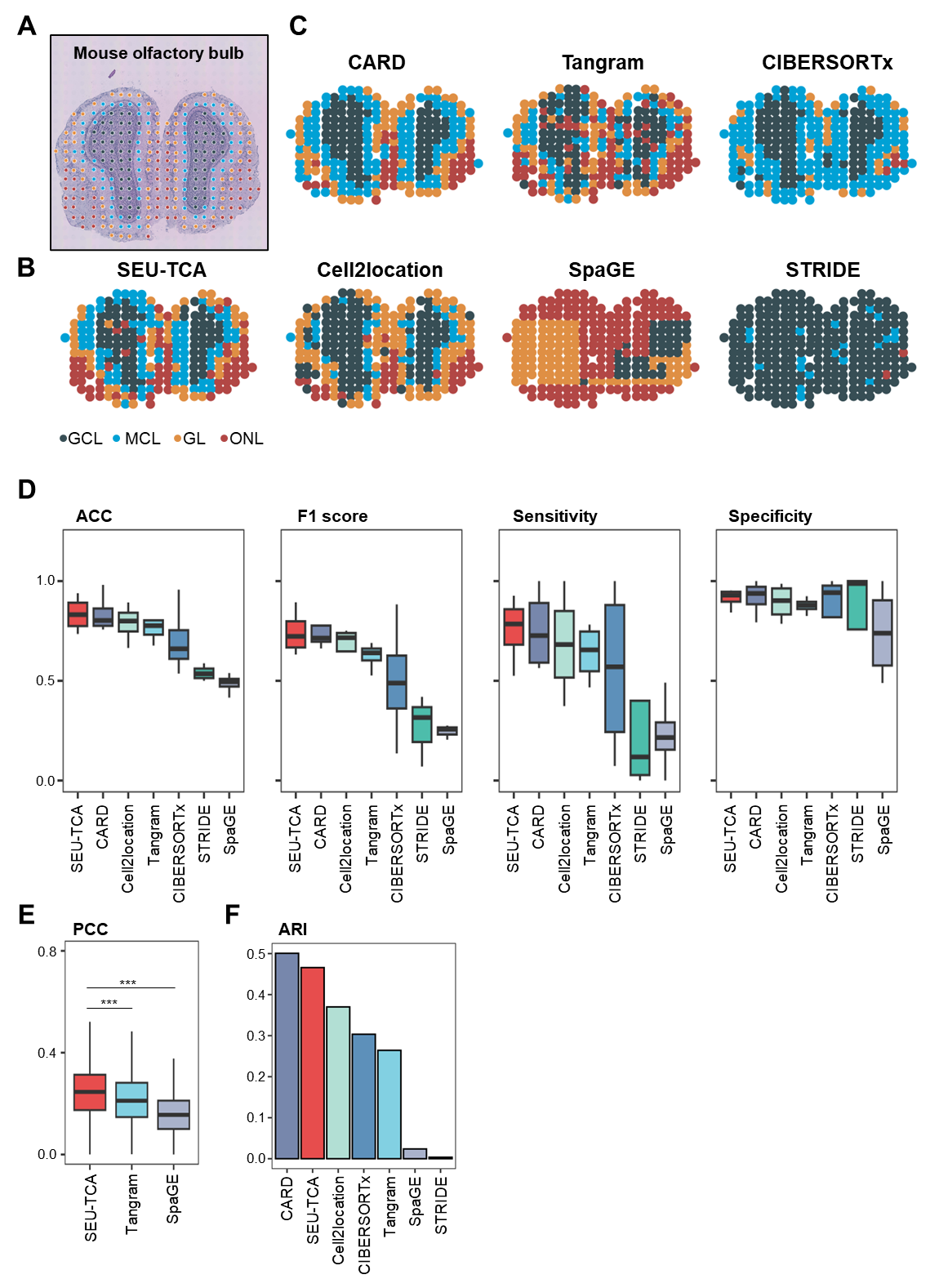
**

**(A)** The base layer shows H&E staining of the mouse olfactory bulb, overlaid with the ground truth annotated by histological structure. **(B)** Dominant cell types inferred using SEU-TCA is shown. **(C)** Dominant cell types inferred using other six methods are shown. **(D)** ACC, F1 score, Sensitivity, and Specificity performances of different methods on the mouse olfactory bulb dataset. Each value represents the comparison between the predicted dominant cell type across different anatomical layers and the ground truth. The methods are ordered based on the median accuracy values, sorted from highest to lowest. **(E)** PCC performance of different methods on the mouse olfactory bulb dataset. Each value represents the correlation between spot-cell pairs predicted by these three methods at the expression level of top 50 genes for each cell type. 95% confidence intervals of mean PCC values for each method were as follows: SEU-TCA ([0.240,0.244]), Tangram ([0.212,0.215]), and SpaGE ([0.149,0.152]). Statistical comparisons were conducted using pairwise t-tests, and p-values were adjusted for multiple testing using the FDR method. Significance levels are denoted by asterisks: ***: p < 0.001. **(F)** ARI performance of different methods on the mouse olfactory bulb dataset. Each value represents the similarity between the inferred dominant cell types and ground truth. The methods are ordered based on the ARI values, sorted from highest to lowest.

**Fig S5. The performance evaluation results of different integration methods on the human pancreatic ductal adenocarcinoma dataset.**

**
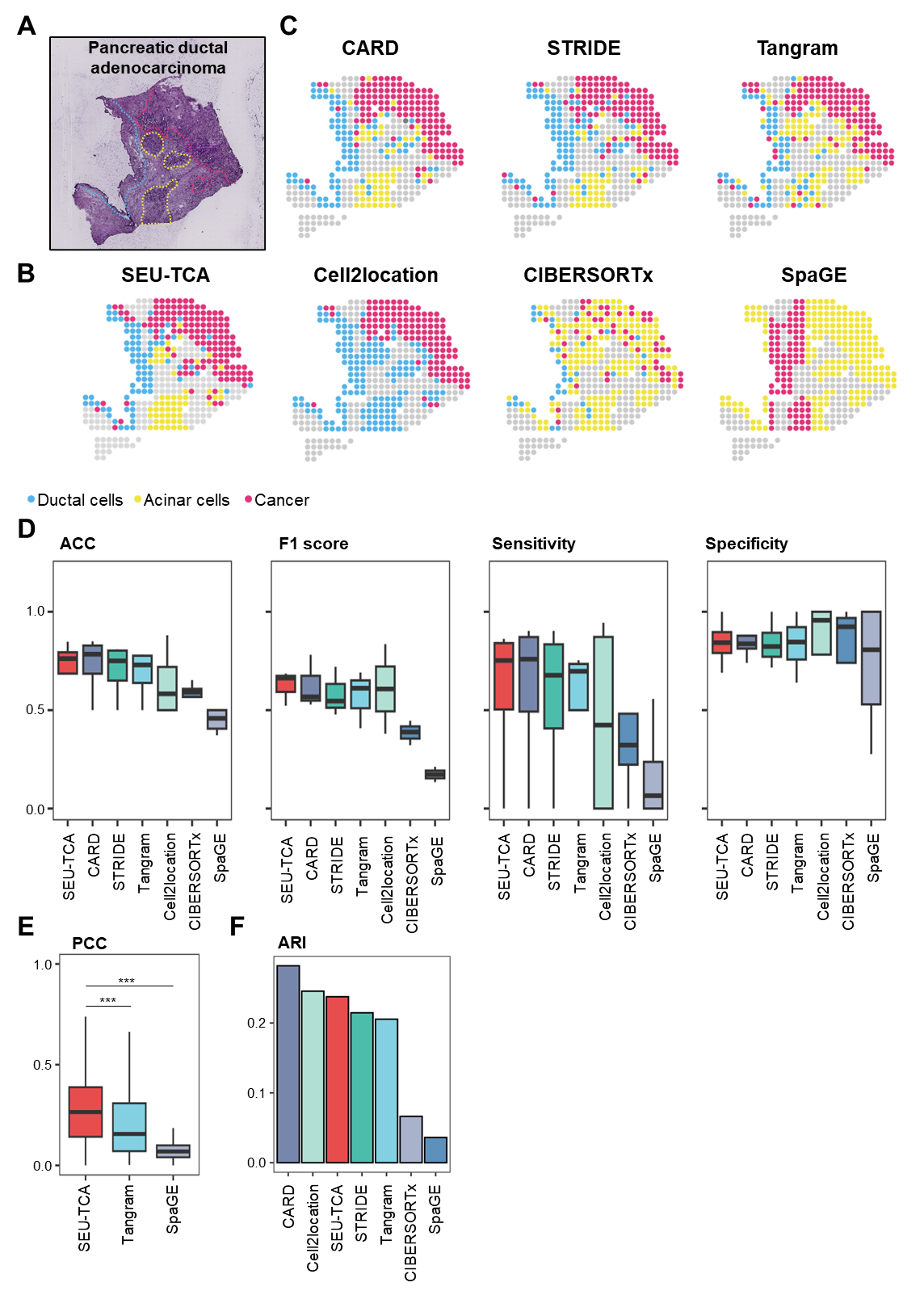
**

**(A)** The base layer shows H&E staining of the human pancreatic ductal adenocarcinoma displaying three regions with well-defined boundaries. **(B)** Dominant cell types inferred using SEU-TCA is shown. **(C)** Dominant cell types inferred using other six methods are shown. **(D)** ACC, F1 score, Sensitivity, and Specificity performances of different methods on the pancreatic ductal adenocarcinoma dataset. Each value represents the comparison between the predicted dominant cell type across different anatomical layers and the ground truth. The methods are ordered based on the median accuracy values, sorted from highest to lowest. **(E)** PCC performance of different methods on the pancreatic ductal adenocarcinoma dataset. Each value represents the correlation between spot-cell pairs predicted by these three methods at the expression level of top 50 genes for each cell type. 95% confidence intervals of mean PCC values for each method were as follows: SEU-TCA ([0.236,0.262]), Tangram ([0.126,0.153]), and SpaGE ([0.040,0.050]). Statistical comparisons were conducted using pairwise t-tests, and p-values were adjusted for multiple testing using the FDR method. Significance levels are denoted by asterisks: ***: p < 0.001. **(F)** ARI performance of different methods on the pancreatic ductal adenocarcinoma dataset. Each value represents the similarity between the inferred dominant cell types and ground truth. The methods are ordered based on the ARI values, sorted from highest to lowest.

**Fig S6. Validation for SEU-TCA model using GEO-seq and scRNA-seq data.**


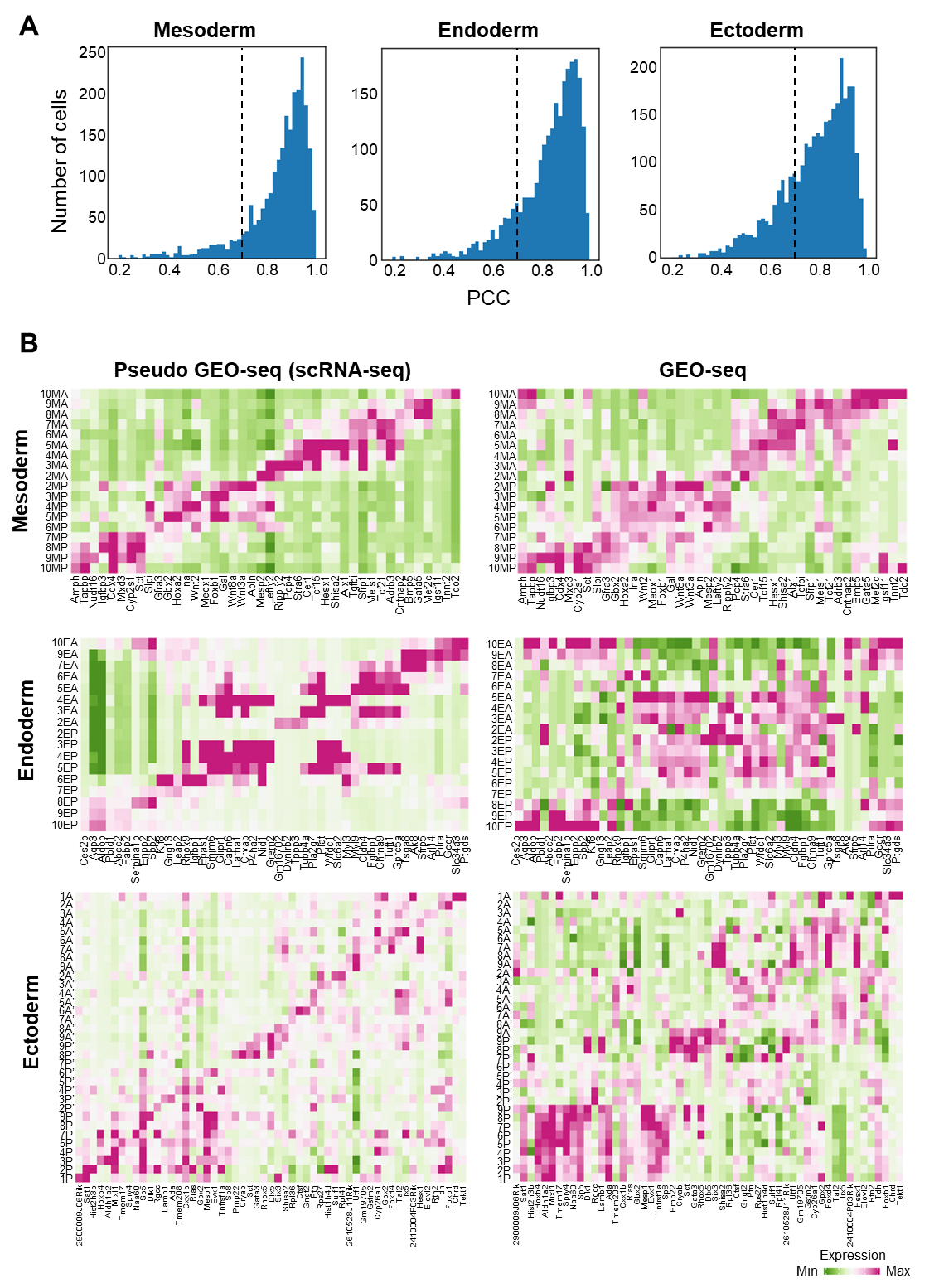


**(A)** Bar charts illustrate the distribution of Pearson correlation coefficient (PCC), which were calculated between spots in the spatial transcriptome and cells in the single-cell transcriptome in shared latent space, for mesoderm (left), endoderm (middle) and ectoderm (right). Bar height denotes the number of cells within different ranges of PCC for each germ layer. Applying a threshold of PCC equal to 0.7, cells are filtered for downstream analysis. **(B)** Method validation was performed using mesoderm (top row) endoderm (middle row) and ectoderm (bottom row) data obtained from E7.5 mouse embryo’s GEO-seq and scRNA-seq datasets. Heatmaps illustrate expression levels of the top three marker genes across the predicted locations for scRNA-seq data.

**Fig S7. The performance evaluation results of different integration methods on E7.5 GEO-seq and scRNA-seq data of the mouse embryo.**


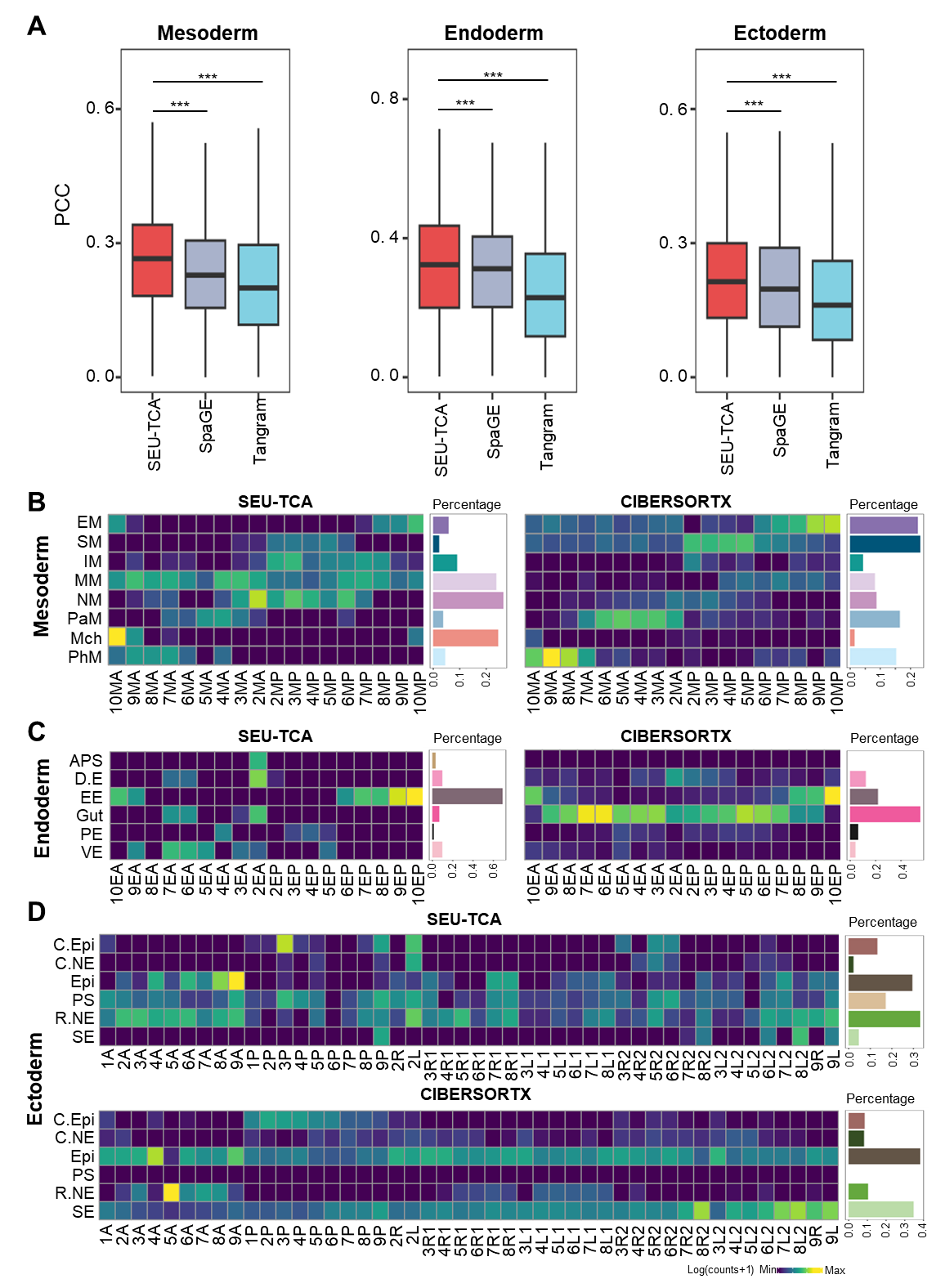


**(A)** PCC performance of different methods on the mesoderm, endoderm, and ectoderm data. Each value represents the correlation between spot-cell pairs predicted by these three methods at the expression level of top 50 genes for each cell type. 95% confidence intervals of mean PCC values for each method were as follows: SEU-TCA (mesoderm [0.254,0.263], endoderm [0.305,0.317], and ectoderm [0.204,0.213]), SpaGE (mesoderm [0.211,0.221], endoderm [0.291,0.303], and ectoderm [0.186,0.195]), and Tangram (mesoderm [0.128,0.142], endoderm [0.097,0.116], and ectoderm [0.118,0.129]). Statistical comparisons were conducted using pairwise t-tests, and p-values were adjusted for multiple testing using the False Discovery Rate (FDR) method. Significance levels are denoted by asterisks: ***: p < 0.001. Deconvolution on the mesoderm **(B)**, endoderm **(C)**, and ectoderm **(D)** GEO-seq data using SEU-TCA and CIBERSORTx. Heatmaps showing the abundance of individual cell types in each spot in GEO-seq data. Rows represent cell types, columns represent spatial locations, and the redder the color, the greater the number of single-cells that fall into that spatial location. The bar plots showing the proportion of cell types predicted by each method in each germ layer.

**Fig S8. Mesodermal data exhibited a distribution along the P-D and A-P developmental axes after SEU-TCA alignment.**


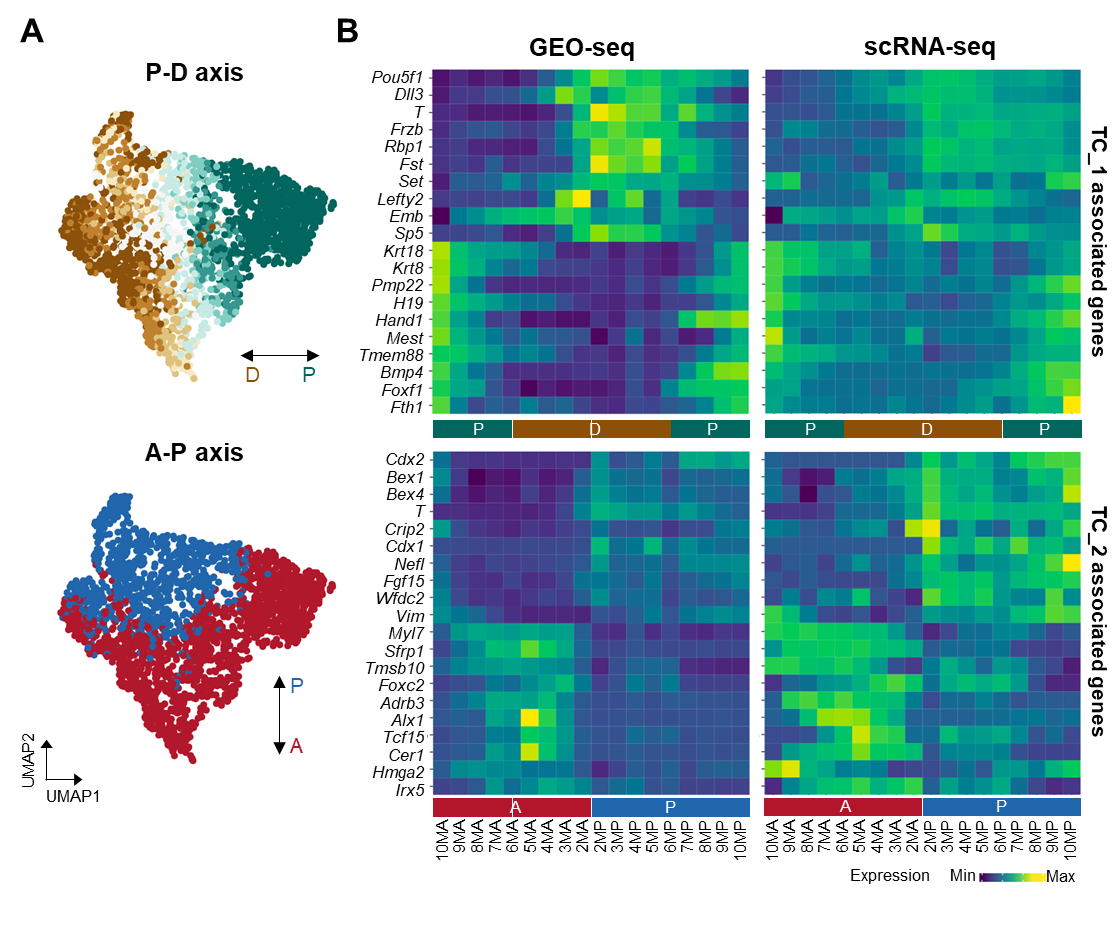


**(A)** UMAP layout for the E7.5 mesodermal cells from Pijuan-Sala et al. is colored by the score of P-D (upper) and A-P (lower) axes predicted by SEU-TCA. Embryonic axes: anterior–posterior, A-P; proximal–distal, P-D. **(B)** Heatmaps illustrate the consistency of expression levels of TC_1 and TC_2 associated genes for the P-D and A-P axes on single-cell data and spatial data, respectively.

**Fig S9. Inference of the spatial locations of cell types for mesoderm.**


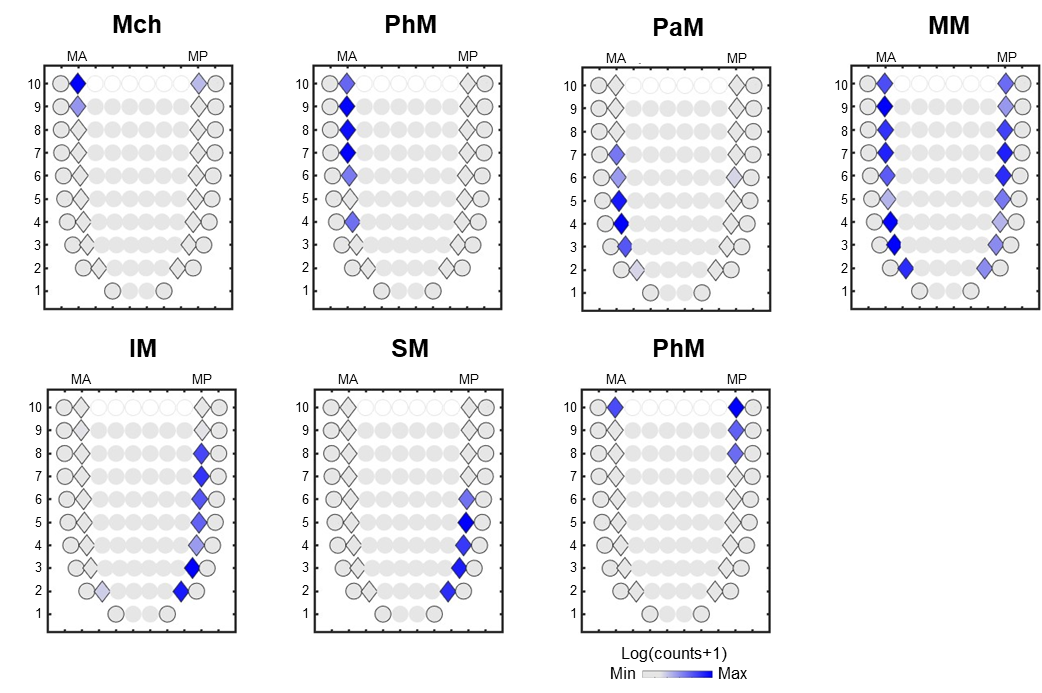


Corn plot showing the spatial pattern of inferred contributions of mesodermal cell types at E7.5.

**Fig S10. Consistency of cell type-specific regulon activity with its gene expression pattern.**


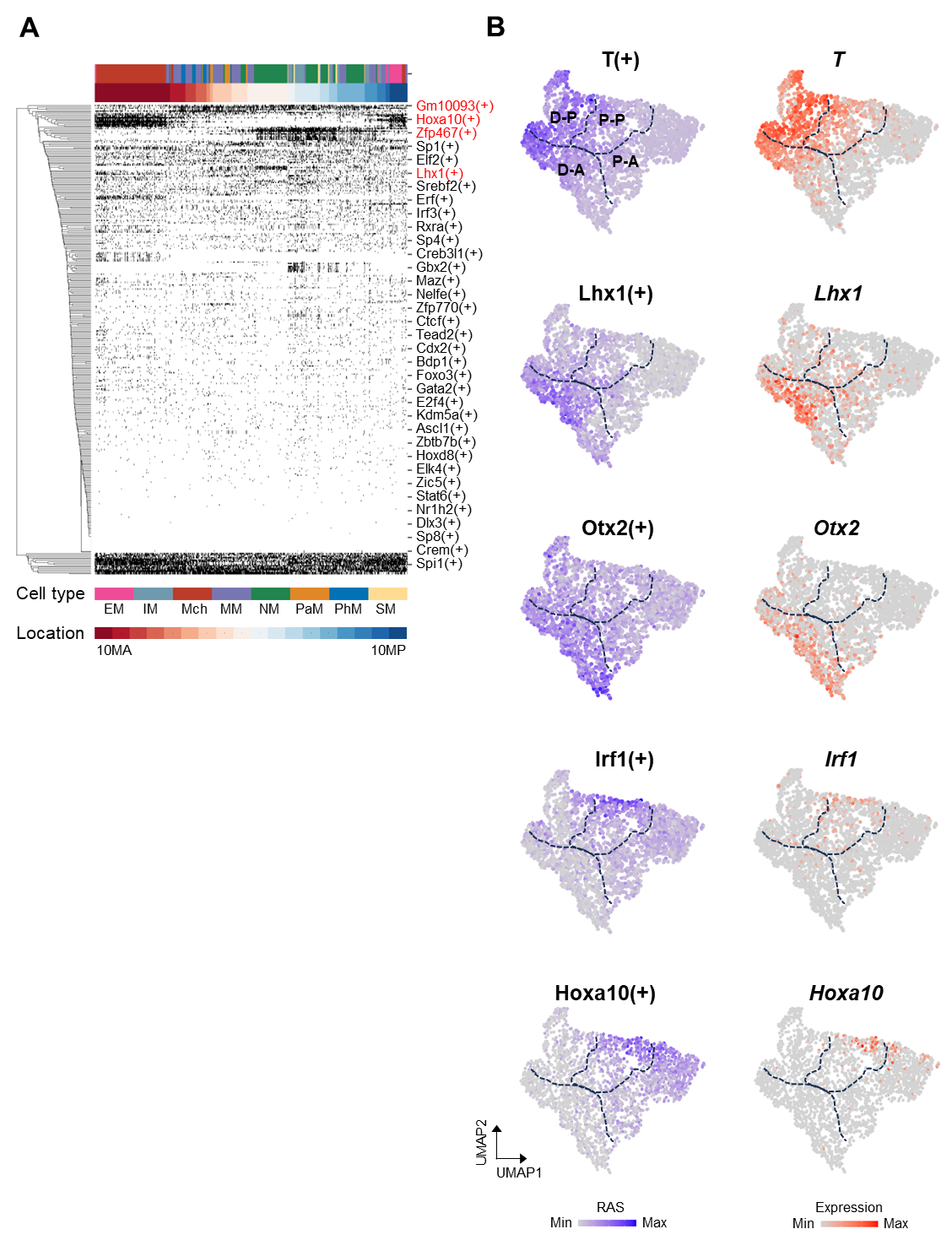


**(A)** Heatmap visualizing the binary regulon activity for each cell is generated from the SCENIC AUC distribution. Black blocks on the heatmap indicate that regulon is active in cells. The top rows represent the signature of E7.5 mesodermal cells from scRNA-seq, indicating both the cell type and spatial location. Single-cells (columns) are ordered by the predicted locations. **(B)** UMAP showing the single-cell resolution pattern of regulon activity (blue) and expression (red) of *T*, *Lhx1*, *Otx2*, *Irf1*, and *Hoxa10* in E7.5 mesodermal scRNA-seq.

**Fig S11. Regulon specificity score for each spatial location.**


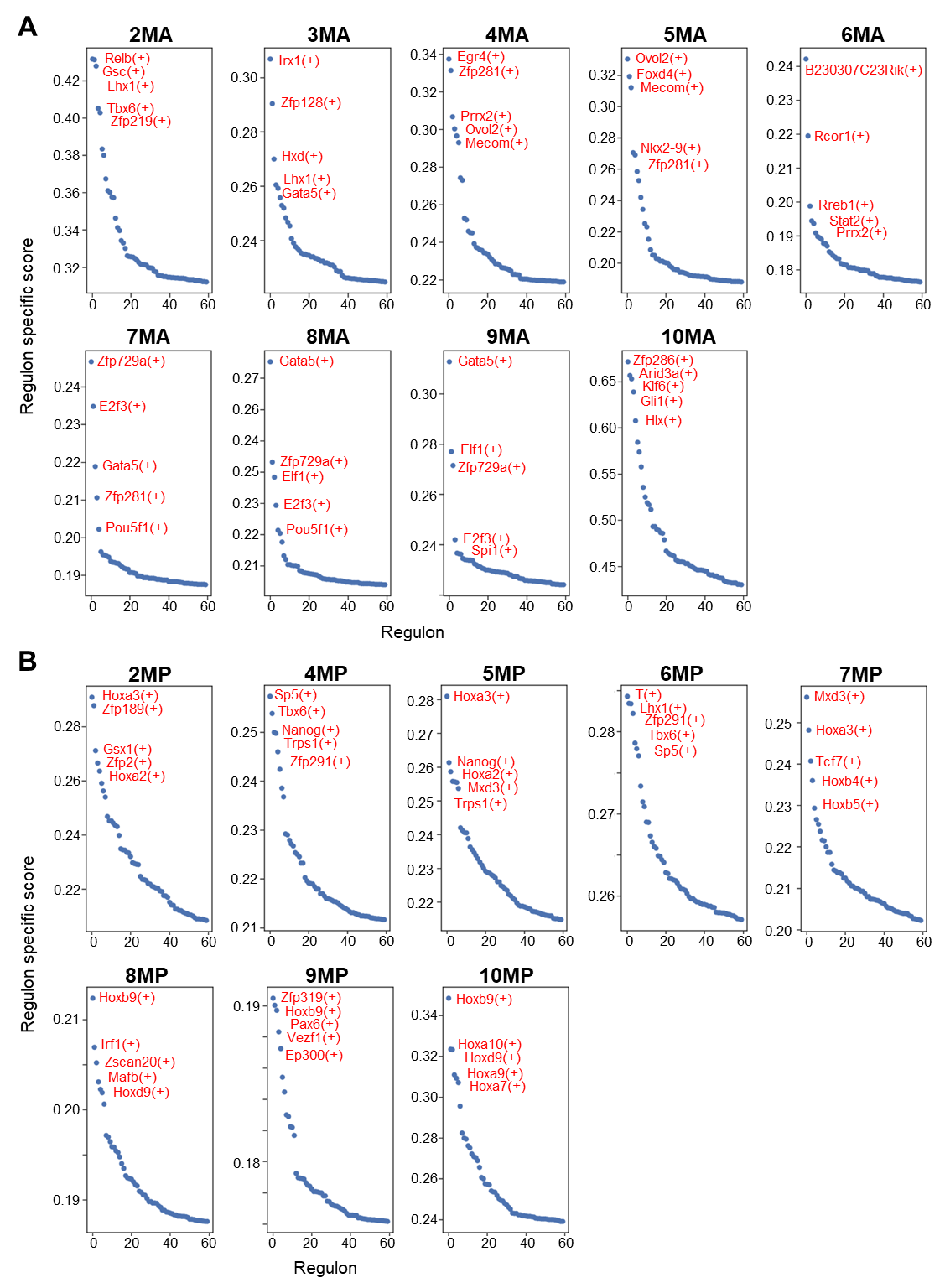


The top five regulons for the anterior location **(A)** and the posterior location **(B)** are highlighted in red and labeled on the plot. The y-axis displays the regulon specificity score.

**Fig S12.** **WOT tracing analysis of JCF/pSHF/aSHF lineage.**

**
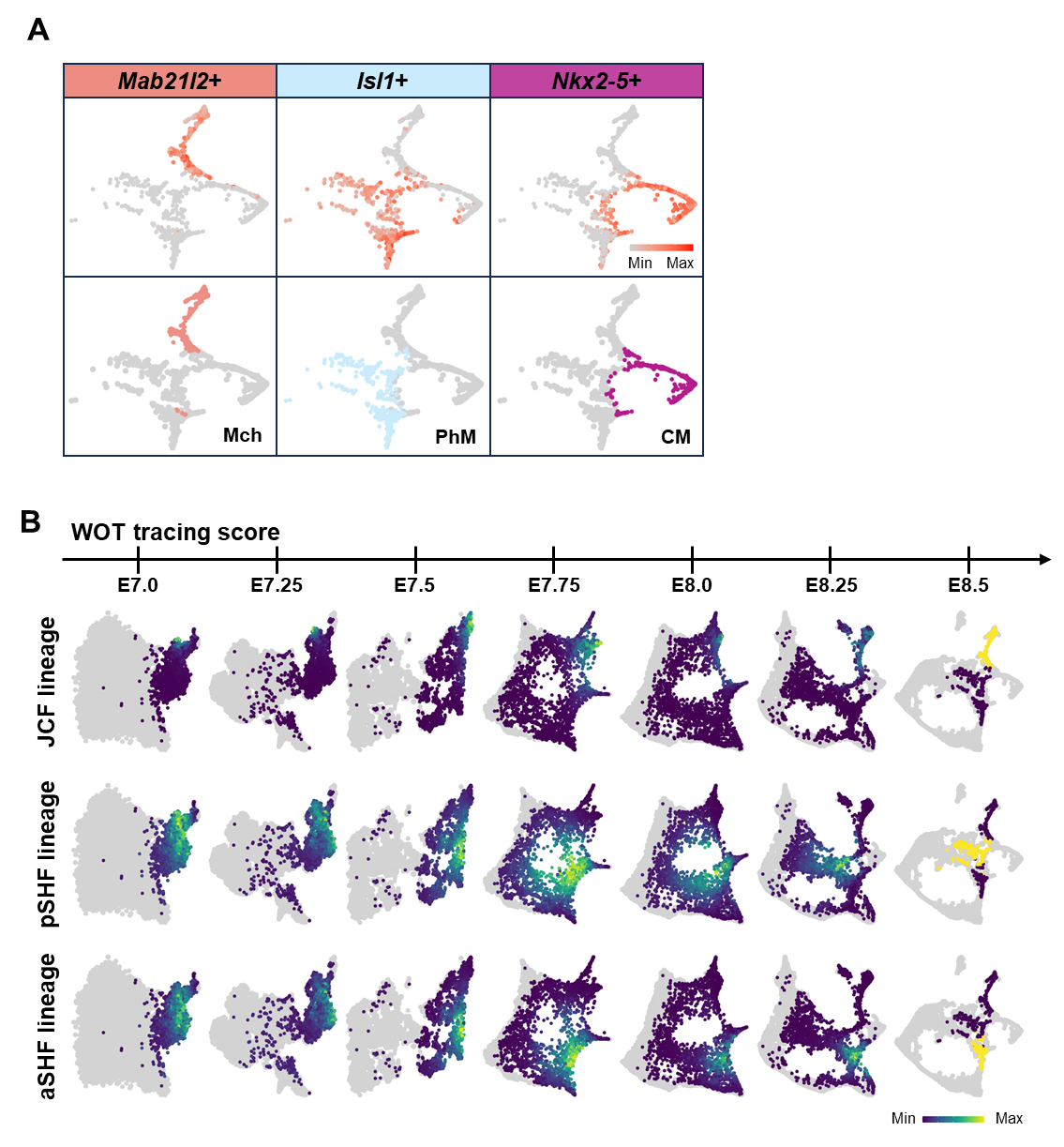
**

**(A)** Selection of cardiac-related cells, including JCF progenitors (Mab21l2-positive Mch) and SHF progenitors (Isl1-positive PhM), as well as mature cardiomyocytes (Nkx2-5-positive CM). **(B)** Time-series tSNE layouts showing the WOT tracing score of JCF/pSHF/aSHF lineage from E7.0 to E8.5. E8.5 JCF/pSHF/aSHF cells were used as trajectory endpoints and traced back to the E7.0, respectively. At each time point, cells of a lineage are selected if WOT score>0.0001.

**Fig S13.** **Markers expression during the mesodermal lineage.**


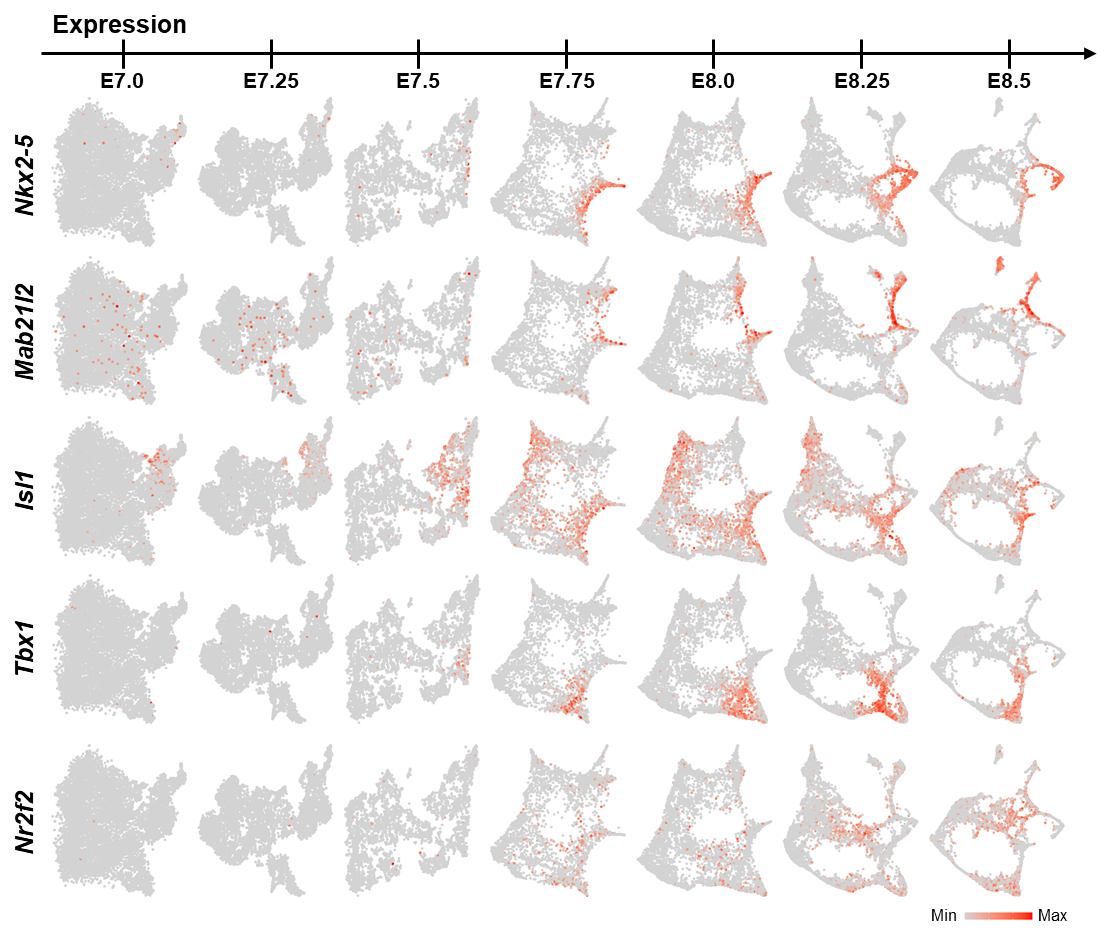


Time-series tSNE layouts showing the expression pattern of the marker of CM (*Nkx2-5*), JCF/FHF (*Mab21l2*), SHF (*Isl1*), aSHF (*Tbx1*), and pSHF (*Nr2f2*) from E7.0 to E8.5.

**Fig S14.** **Specific spatial regulon in JCF/aSHF/pSHF lineages.**


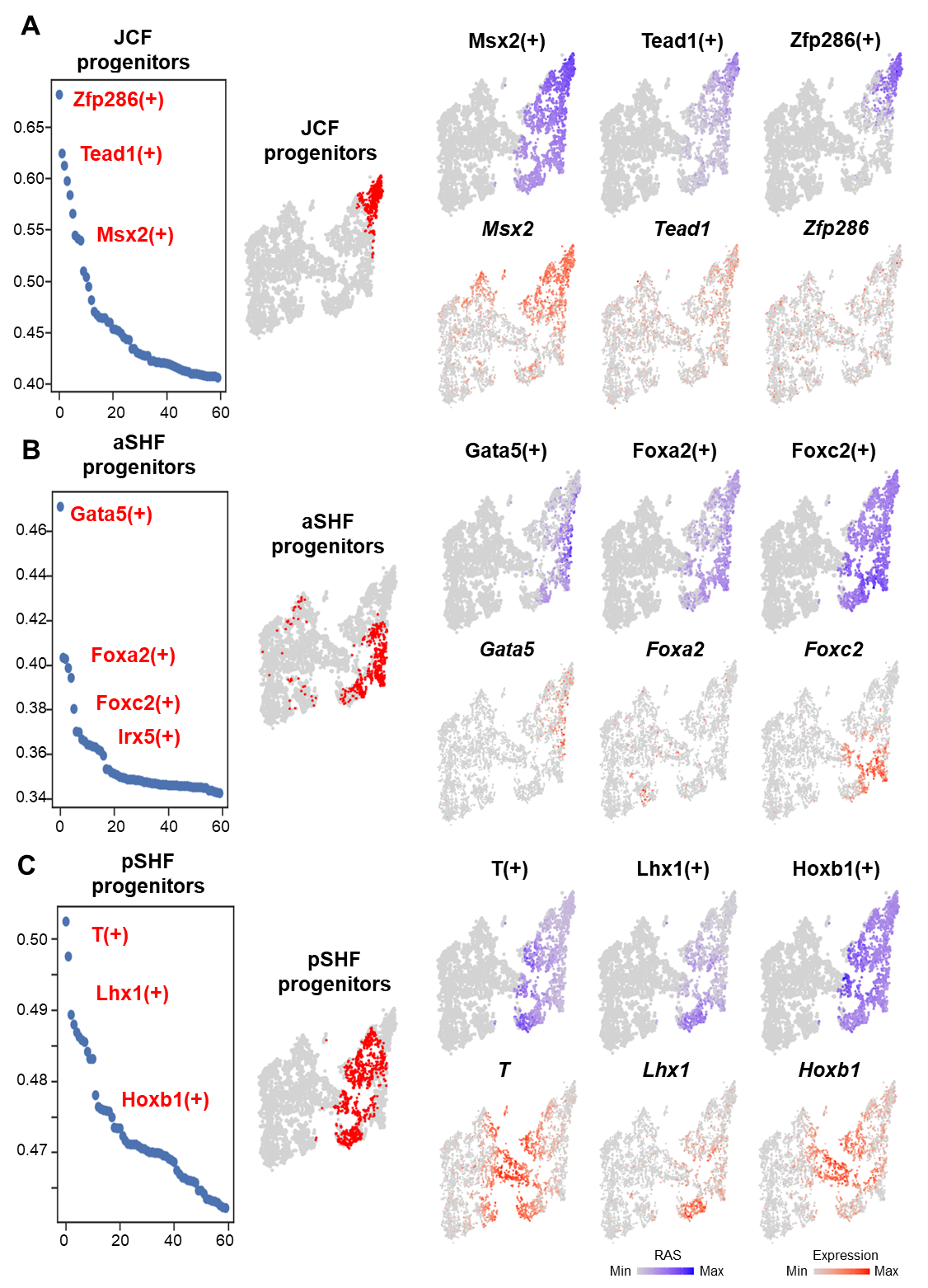


The scatter plots on the left showing the top regulons for the JCF progenitors **(A)**, aSHF progenitors **(B)**, and pSHF progenitors **(C)** are labeled on the plot. The y-axis displays the regulon specificity score. UMAPs on the middle highlight the JCF progenitors **(A)**, aSHF progenitors **(B)**, and pSHF progenitors **(C)** in E7.5 mesodermal scRNA-seq. UMAPs on the right showing the single-cell resolution pattern of regulon activity (blue) and expression (red) of target genes in E7.5 mesodermal scRNA-seq.

**Fig S15.** **Expression patterns of *Irx1*/*3*/*5* in the aSHF lineage.**


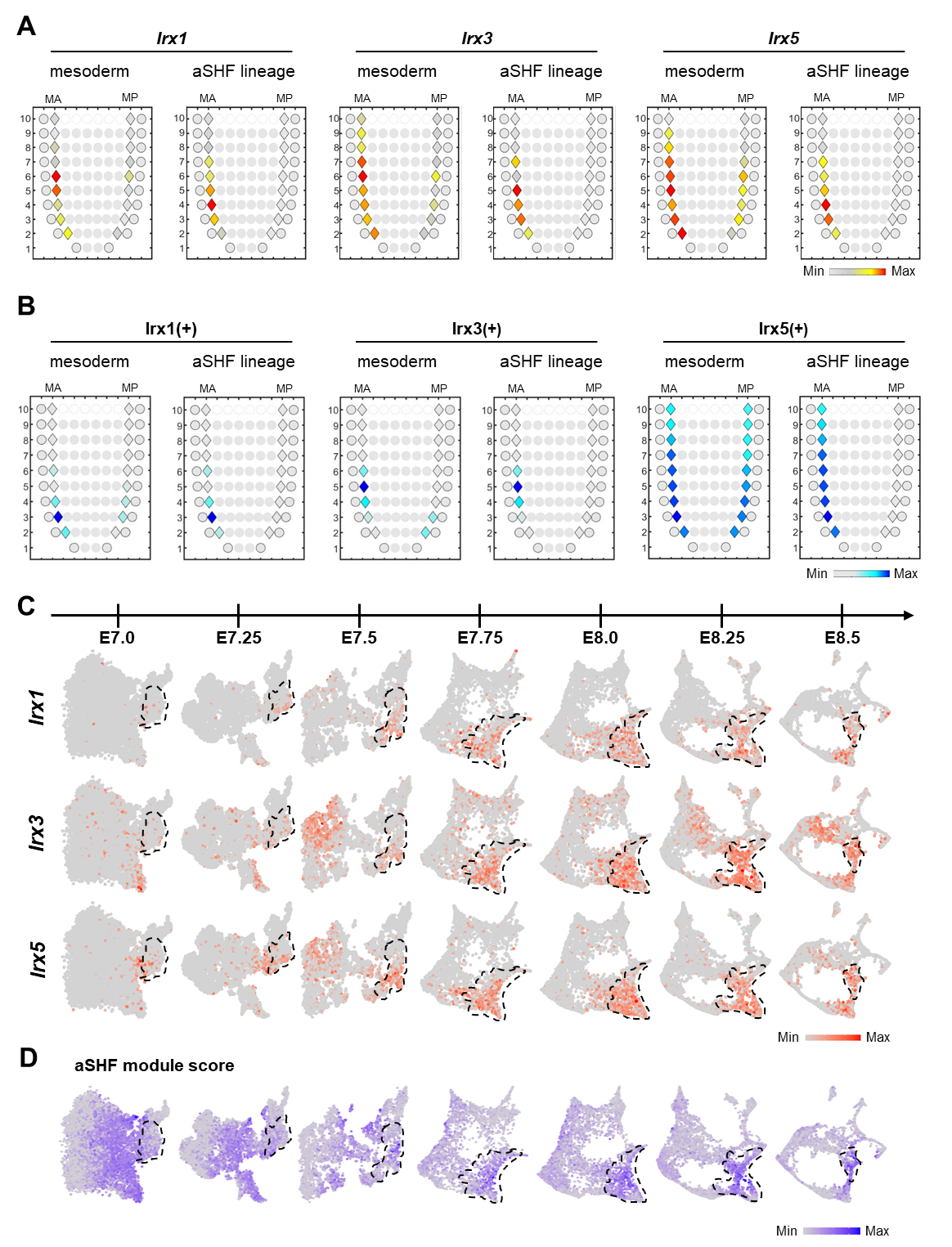


**(A)** Corn plots showing the spatial pattern of expression of *Irx1*, *Irx3,* and *Irx5* in mesoderm and aSHF lineage at E7.5, respectively. **(B)** Corn plots showing the spatial pattern of regulon activity of *Irx1*, *Irx3,* and *Irx5* in E7.5 mesodermal GEO-seq. **(C)** Time-series tSNE layouts showing the expression pattern of *Irx1*, *Irx3*, and *Irx5* during aSHF lineage from E7.0 to E8.5. **(D)** aSHF module scores for a set of aSHF marker genes (e.g., *Isl1*, *Tbx1*, *Fgf8*, *Tcf21*) across all cells using Seurat's ‘AddModuleScore’ function.

**Fig S16.** **Generation of the *Irx1*-lineage and *Irx1* CKO mice.**


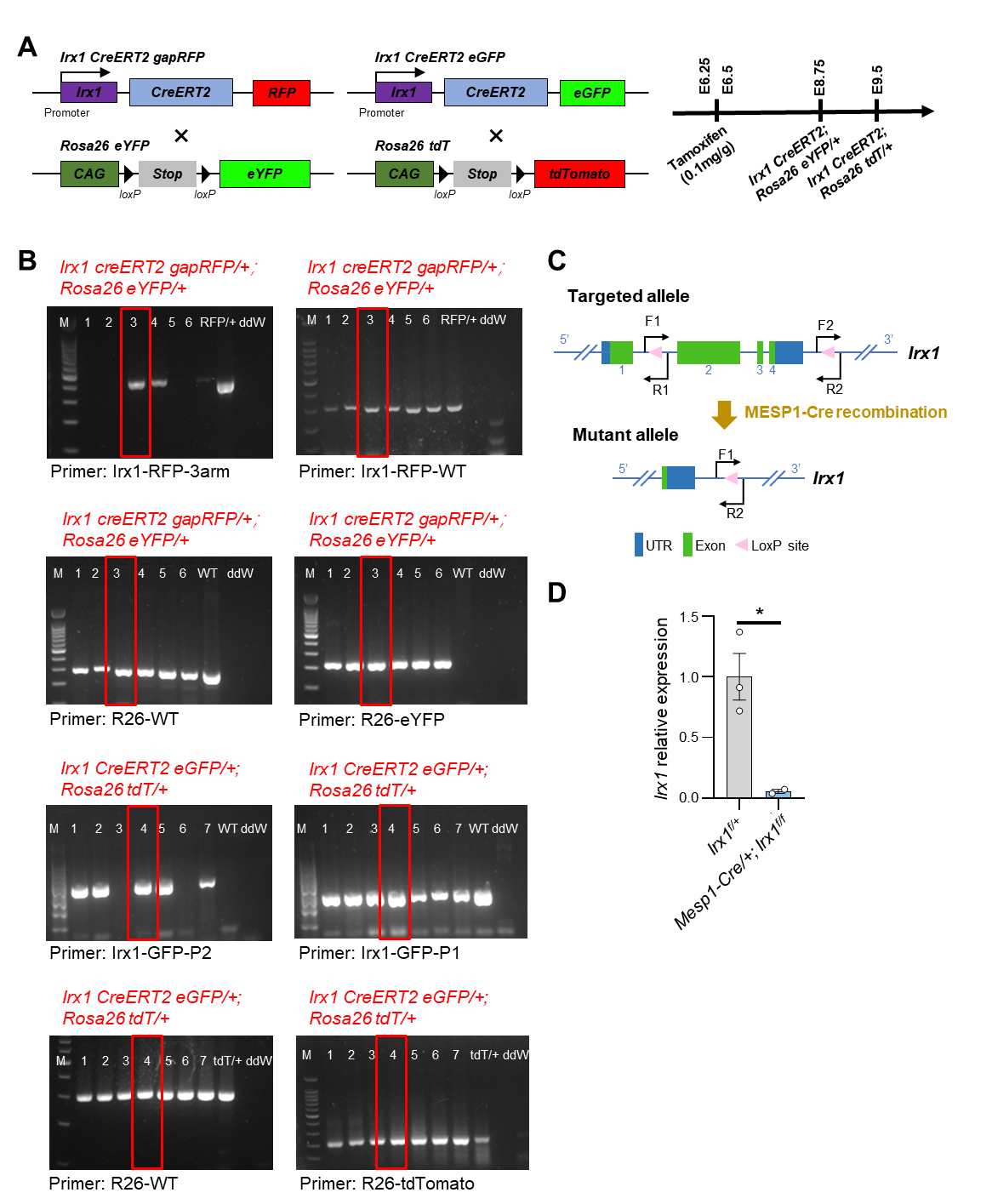


**(A)** Schematic of the mouse model used for lineage tracing of Irx1-positive cells. The top panel illustrates the generation of *Irx1-CreERT2* mice, in which the CreERT2 recombinase is inserted into the endogenous *Irx1* locus, allowing for tamoxifen-inducible Cre recombination specifically in Irx1-positive cells. In the presence of CreERT2, the Stop cassette in *Rosa26-eYFP or* *Rosa26-tdTomato* reporter allele is excised, leading to the expression of the eYFP or tdTomato fluorescent protein under the control of the ubiquitous Cag promoter. The timeline on the right indicates the experimental timeline for tamoxifen administration and subsequent analysis. **(B)** Genotyping analysis of *Irx1-CreERT2-gapRFP*, *Rosa26-eYFP, Irx1-CreERT2-eGFP* and *Rosa26-tdTomato* mice. The panels showing PCR amplification results for the identification of *Irx1-CreERT2-gapRFP*, *Rosa26-eYFP, Irx1-CreERT2-eGFP* and *Rosa26-tdTomato* alleles in mouse genomic DNA. **(C)** Mating strategy for the generation of *Irx1* mutant mice. Mice carrying loxP-flanked (floxed) *Irx1* alleles are crossed with mice expressing Cre recombinase under the control of the *Mesp1* promoter. A detailed examination was conducted at E13.5. **(D)** Quantification of *Irx1* transcription by qRT-PCR in E9.5 *Irx1^f/+^* and *Mesp1-Cre; Irx1^f/f^* embryos. The sample size (n) represents the number of embryos: n=3 for the *Irx1^f/+^* group and n=2 for the *Mesp1-Cre; Irx1^f/f^* group. *P*-values were calculated using the Wilcoxon rank-sum test. Error bars are SEM. *p < 0.05.
